# The Clinical Application of Neoantigens in Esophageal Cancer

**DOI:** 10.3389/fonc.2021.703517

**Published:** 2021-07-27

**Authors:** Yi-Min Gu, Yue Zhuo, Long-Qi Chen, Yong Yuan

**Affiliations:** ^1^Department of Thoracic Surgery, West China Hospital of Sichuan University, Chengdu, China; ^2^West China School of Medicine, Sichuan University, Chengdu, China

**Keywords:** esophageal cancer, immunotherapy, neoantigen, cancer vaccine, adoptive cell therapy

## Abstract

Esophageal cancer (EC) is a common malignant tumor with poor prognosis, and current treatments for patients with advanced EC remain unsatisfactory. Recently, immunotherapy has been recognized as a new and promising approach for various tumors. EC cells present a high tumor mutation burden and harbor abundant tumor antigens, including tumor-associated antigens and tumor-specific antigens. The latter, also referred to as neoantigens, are immunogenic mutated peptides presented by major histocompatibility complex class I molecules. While current genomics and bioinformatics technologies have greatly facilitated the identification of tumor neoantigens, identifying individual neoantigens systematically for successful therapies remains a challenging problem. Owing to the initiation of strong, specific tumor-killing cytotoxic T cell responses, neoantigens are emerging as promising targets to develop personalized treatment and have triggered the development of cancer vaccines, adoptive T cell therapies, and combination therapies. This review aims to give a current understanding of the clinical application of neoantigens in EC and provide direction for future investigation.

## Introduction

EC ranks as the seventh most common malignant tumor and the sixth leading cause of cancer-related death worldwide. An estimated 572,000 new cancers of EC and 509,000 deaths occurred in 2018 (1). To date, multidisciplinary therapy involving neoadjuvant chemoradiotherapy followed by surgery has formed the standard treatment for local advanced EC (2, 3). However, only 29% of patients who undergo resection after chemoradiotherapy show a pathological complete response, and the incidence of grade 3 or 4 treatment-related adverse events during chemoradiotherapy are common (2). The long-term survival for patients with locally advanced EC remains unsatisfactory despite therapeutic improvements (4).

Immunotherapy, including immune checkpoint blockade (5, 6), cancer vaccination (7), and adoptive T cell therapy (8), has been explored as a novel strategy for improving survival outcomes of EC patients recently. Immune checkpoints on the surface of T cells are receptors that are crucial for preventing autoimmune damage to healthy tissues. However, tumor cells exploit immune checkpoint inhibition to evade the immune response (9, 10). Immune checkpoint blockade therapy has dramatically changed the treatment of melanoma and advanced non-small-cell lung cancer (11, 12). Several randomized clinical trials have shown that immune checkpoint blockade is a promising prospect for the treatment of EC (5, 13, 14).

It is known that accumulation of genetic alterations can result in cancer development. Neoantigens, referred to as immunogenic mutated peptides in tumor cells, are presented by major histocompatibility complex class I molecules and stimulate a strong cytotoxic T cell–mediated immune response (15).

EC appears to be associated with a high tumor mutation burden and neoantigen load compared with esophageal adenocarcinoma (16, 17). Due to their strong tumor-specific immunogenicity, neoantigens are emerging as a promising target for tumor immunotherapy. Immune checkpoint blockade achieves tumor control by releasing immune inhibition, while neoantigens aim to elicit the immunogenicity of tumors and trigger cytotoxic T cell responses, thereby improving antitumor efficacy (18).

In this review, we focus on the clinical application of neoantigens and highlight advances in the use of neoantigens in cancer vaccines, adoptive T cell therapy, and combination therapy for EC, and we discuss the present barriers and strategies.

## Cancer Vaccines

Traditional cancer vaccines mainly use tumor-associated antigens, which are also expressed in normal human tissue. Owing to their high tumor-specific and exceptional immunogenicity, tumor neoantigens are ideal targets for cancer vaccine design (19, 20). Neoantigen vaccines, a new type of tumor immunotherapy, can induce a strong, specific immune response and elicit stable therapeutic effects.

New York esophageal squamous cell carcinoma 1 (NY-ESO-1) is known as a member of the testis cancer gene family with high immunogenicity. Oshima et al. screened 1,969 patients with various cancers and identified serum NY-ESO-1 antibody as a tumor-specific biomarker for EC (21). Subsequently, Wada et al. performed a phase I clinical trial using NY-ESO-1 protein as a cancer vaccine for eight patients with advanced EC (22). Antibody, CD4+, and CD8+ T cell responses were recognized in 87.5% (7/8), 87.5% (7/8), and 75.0% (6/8) of patients, respectively. Nevertheless, disease progression was eventually observed in all patients after vaccination for various reasons, such as primary tumor growth or paratracheal or abdominal lymph node metastases.

In a phase I dose-escalation trial of a two-dose cohort, cholesteryl pullulan (CHP)-NY-ESO-1 vaccine was confirmed to be safe and to induce immunogenicity in patients with advanced metastatic EC. The 200 μg dose cohort elicited stronger immune responses and displayed better survival outcomes than the 100 μg dose cohort (23). In addition, a phase II comparative study of CHP-NY-ESO-1 vaccine was performed in NY-ESO-1-expressing patients with esophageal squamous cell carcinoma who underwent neoadjuvant chemotherapy followed by surgery. No significant difference in survival benefit was noted between the CHP-NY-ESO-1 vaccine group and untreated controls (24).

Several studies have shown that the neoantigen vaccine can successfully induce cytotoxic T cells and trigger immune effects (25–27). However, sufficient and persistent immune effects are difficult to achieve with antitumor vaccines (25, 26). Rosenberg et al. suggested tumor cells possibly possess multiple immune escape mechanisms, such as insufficient tumor antigen expression, the tumor produces local immunosuppressive factors, T cells are “tolerized”, or there is downregulation of T cell receptor signal transduction, among other things (28).

Compared with traditional peptide vaccines, mRNA vaccines are a genetic vaccine that might stimulate an effective immune response. Notably, Forghanifard et al. were the first to report that a novel chimeric mRNA-loaded dendritic-cell (DC) vaccine can elicit an effective immune response and induce cytotoxicity against EC *ex vivo*. Although this was a preclinical study, it provided a new approach to use DC-based vaccines for EC immunotherapy (29).

## Adoptive T Cell Therapy

Tumor-specific neoantigens can also serve as promising targets for adoptive cell therapy. This involves patient T cells that have been genetically engineered to target a tumor cell surface antigen. The current strategies mostly use antigen-specific T cell receptor (TCR) gene-engineered T cells, chimeric antigen receptor (CAR) T cells, or tumor-infiltrating lymphocytes (TIL). These therapies have yielded remarkable tumor responses in clinical trials of hematological tumors and high mutation tumors (30, 31). However, these therapeutic options have limited therapeutic effect or are poorly understood in solid tumors.

Kageyama et al. conducted a first-in-man trial using adoptive cell therapy of MAGE-A4 T cell receptor gene-transduced lymphocytes in patients with MAGE-A4-expressing EC. Results revealed that T cell receptor gene-engineered T cells can be detected in the peripheral blood of all patients at the initial administered level for 1 month. Notably, transferred T cells in five patients persisted for more than 5 months. However, the antitumor effect was limited. Despite the long persistence of transferred T cells in blood, disease progression occurred in 70% (7/10) of patients after treatment. It is noteworthy that 30% (3/10) of patients with minimal tumor lesions had progression-free survival for 27 months (32). These findings suggest that adoptive T cell therapy may provide better survival outcomes in patients with early-stage tumors compared with those with advanced or recurrent EC. An explanation may be that various mechanisms underlie the benefits of adoptive T cell therapy. Another explanation might be cross-reactivity to corresponding wild-type peptide sequences in the identification of neoantigens. A recent study reported the establishment of a protocol for effective construction of neoantigen-specific TCR T cells to improve therapeutic outcomes (33).

Advances in genomics and bioinformatics have accelerated the emerging technology of CAR T cells, which transformed the field of adoptive T cell therapy. To our knowledge, no clinical trial using neoantigen target CAR T cell therapy in EC patients has been published. It has been reported that CAR T cells targeting EphA2 receptors stimulated antitumor activity of EC cells in a preclinical trial (34). The only clinical trial of CAR T cell therapy for EC, targeting the EpCAM adhesion molecule, is ongoing (NCT03013712). Both EphA2 and EpCAM are tumor-associated antigens that are overexpressed in patients with esophageal squamous cell carcinoma but are not tumor-specific antigens (34–36).

TIL is another candidate for T-cell therapy. Several clinical studies have demonstrated successful adoptive TIL therapy for solid tumors such as melanoma and ovarian cancer (37–40). In EC, TIL positivity is associated with better survival outcomes (41). Tan et al. used TIL to construct neoantigen-specific TCR T cells for esophageal squamous cell cancer and identified antitumor activity *in vivo* and *in vitro* (42). Due to this study only enrolling one patient, it is difficult to comprehensively assess the efficacy of adoptive TIL therapy. We recommend future studies enroll larger populations. To date, a clinical trial of adoptive TIL transfer therapy for EC has not been reported.

## Combination Therapy

Although neoantigen-specific cancer vaccines or adoptive T cell therapy can elicit specific immune responses, tumor cells can escape from the immune system through multiple mechanisms (43–45). Additionally, the tumor microenvironment is rather complex and quite different from an *in vitro* milieu. Components derived from a neoantigen-enriched tumor microenvironment could suppress the function of effector T cells (46, 47). Therefore, combinations of neoantigen immunotherapy and different modes of treatment are now much more attractive.

Studies found that cancer vaccines could stimulate the proliferation of specific T cells; however, the expression of the programmed death ligand PD-L1 correspondingly increased (48, 49). This might suggest that cancer vaccines combined with a PD-L1 inhibitor or a PD-1 receptor inhibitor may be a possible therapeutic strategy. In a preclinical trial, Ishihara et al. tested a combination therapy of CHP-NY-ESO-1 cancer vaccine and an anti-PD-1 monoclonal antibody and induced significant tumor suppression (*P* = 0.029) compared with the no-treatment group (27). This combination therapy is a promising strategy and merits future attention.

Beyond this, it has been reported that a tumor-specific vaccine combined with immune checkpoint therapy can lead to a more efficient response than monotherapy in pancreatic and prostate cancer in preclinical comparative studies (50, 51). A neoantigen-specific cancer vaccine combined with adoptive T cell therapy has also been successfully used to achieve an antitumor response. Kageyama et al. conducted a phase I clinical trial of MAGE-A4 peptide vaccinations combined with TCR gene-engineered T cell in EC. Persistence of tumor-specific reactivity was detected although tumor regression was not observed.

Chemotherapy and radiation are important components in the treatment arsenal for EC patients. Several studies have confirmed that chemotherapy or radiotherapy can stimulate tumors to release antigens and induce the production of tumor antigen-specific effector cells in tumor tissue (52, 53). In EC, a phase I clinical trial of a multiple-epitope peptide vaccine combined with chemoradiation therapy has successfully been performed. Notably, six of 11 patients achieved complete response, and four patients with complete response experienced long-term survival benefit (54). Approaches to integrate chemoradiotherapy with immunotherapy provide novel therapeutic strategies and are now in the ascendance.

## Summary and Future Directions

**Figure 1:**
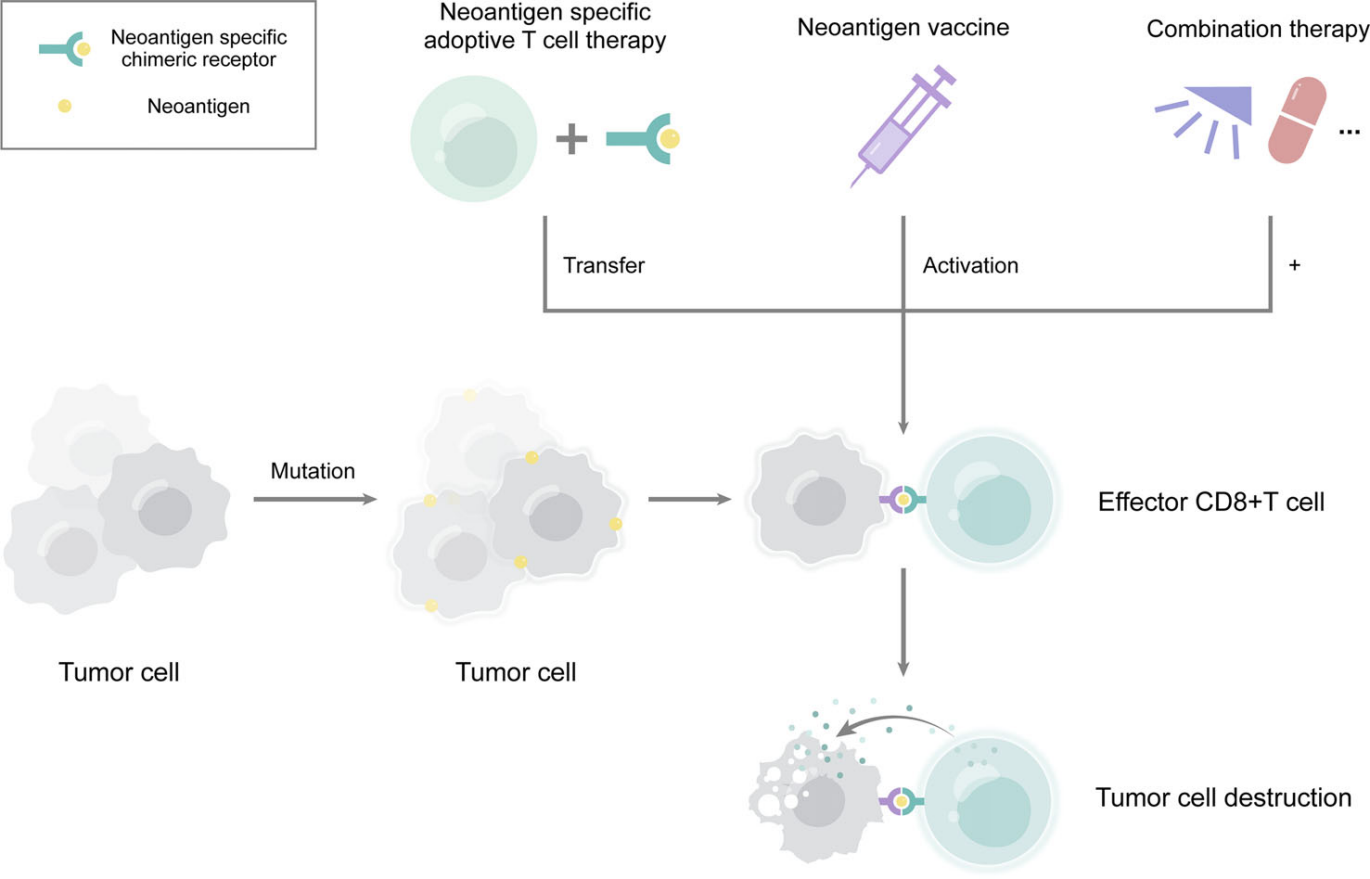
The major clinical application of neoantigens in esophageal cancer. Neoantigens derived from mutations in tumor cells. They can serve many purposes, including neoantigen vaccination, neoantigen specific adoptive T cell therapy, and combination therapy. Combinations of neoantigens with radiotherapy, chemotherapy, or immune checkpoint inhibitor therapy may lead to a better effect. Neoantigens induce the production of effector CD8+ T cells, thereby eliminating tumor cells.

This review summarized the trials with neoantigens for EC or solid tumors including EC (**Table 1**) and the major types of neoantigens in EC with clinical application (**Figure 1**). Compared with immunotherapy based on tumor-related antigens, neoantigen-specific cancer vaccines or adoptive T cell transfer can drive specific immune responses and reduce adverse effects on normal tissue. However, effective antitumor activity and even survival benefit are currently not readily achievable with neoantigen monotherapy (55, 56). The maturation of technologies identifying non-synonymous mutations and evaluating the immunogenicity of mutated peptides will determine the success of future clinical applications (15, 57). Although substantial challenges lie ahead on the road to enabling neoantigen-specific effector T cells to completely eliminate tumor cells, recent advances in whole exome sequencing and bioinformatics technology will greatly facilitate the journey (58–60). There are three major strategies for the selection of candidate neoantigens—*in silico* peptide prediction, mass spectrometry-based immunopeptidomics, and whole-exome sequencing database list (15).

**Table 1:** List of trials with neoantigens for EC or solid tumors including EC.

	Target	Sponsor	Phase	Sample size	Trial identification	Outcome summary
Peptide vaccine	CHP-NY-ESO-1	ImmunoFrontier, Inc.	I	25	NCT01003808	The safety and immunogenicity were confirmed
	CHP-NY-ESO-1	Japan Agency for Medical Research and Development	II	54	UMIN000007905	DFS in 2 years: 56 *vs.* 58% in the vaccine arm and control arm; OS in 2 years: 76 *vs.* 79%, respectively
	HLA-A*2402	Shionogi & Co., Ltd	I	15	UMIN000023324	Cytotoxic T cell response was induced in all patients
Adoptive T cell therapy	MAGE-A4	Mie University	I	10	UMIN000002395	3 patients remained free from PD, survived for more than 27 months
	MAGE-A4	Tianjin Medical University Cancer Institute and Hospital	I	15	NCT01694472	N/A
	MAGE-A4	Adaptimmune	I	52	NCT03132922	Ongoing
	NY-ESO-1	Shenzhen Second People’s Hospital	I	36	NCT02457650	N/A
	NY-ESO-1	University Health Network, Toronto	I	22	NCT02869217	Ongoing
Combination therapy	CHP-NY-ESO-1	ImmunoFrontier Inc.	I	26	UMIN000008006	8 patients had SD
	HLA-A*2402	Teikyo University	I	11	NCT00632333	6 patients of CR and 5 patients of PD were observed

EC, esophageal cancer; CHP, cholesteryl pullulan; NY-ESO-1, New York esophageal squamous cell carcinoma 1; MAGE-A4, melanoma-associated antigen A4; HLA, human lymphocyte antigen; DFS, disease-free survival; OS, overall survival; CR, complete response; SD, stable disease; PD, progressive disease; N/A, not available.

Better understanding the mechanisms of resistance to immunotherapy may enable the development of new strategies to improve clinical outcomes. While tumor cell intrinsic factors for resistance of immunotherapy remain poorly understood, published evidence points to the possibility of alterations in interferon (IFN)-gamma signaling pathways and absence of antigen presentation (61). It is already documented that IFN-gamma is the primary cytokine involved in the recognition and elimination of mutant cells in the classical signaling pathway (62). However, tumor cells could escape this effect by upregulating inhibitory ligands that impede the response of T cells or by inducing mutations in the IFN-gamma signaling pathway, resulting in immune evasion (63, 64). In addition, neoantigens or immunogenic mutated peptides are processed and presented on human leukocyte antigen (HLA) molecules of cancer cells. This observation raises the possibility that resistance to tumor-infiltrating lymphocyte adoptive cell transfer therapy could be mediated through silenced HLA molecules (65).

Tumor cell extrinsic factors may also contribute to resistance. In addition to tumor cells and immune cells, as well as the surrounding stroma, abundant immunosuppressive cells, including T regulatory cells, myeloid-derived suppressor cells, and tumor-associated macrophages, in the tumor microenvironment have important roles in the activity of cytotoxic T cells (66, 67). Spranger et al. showed that tumor immune escape is associated with a mechanism whereby a cancer vaccine could elicit T cell activation and upregulate regulatory T cells simultaneously (68). Moreover, Eil et al. found that tumor necrosis releases intracellular potassium ions and blocks the ionic checkpoint, resulting in inhibition of the effector T cell response (69). Hence, the process of tumor immunogenicity and immune escape involves multiple mechanisms, and there remains much to explore in future research.

To overcome the resistance of immunotherapy, contemporary approaches primarily focus on the combination therapy of neoantigen-specific immunotherapy and traditional therapy. As described previously, chemotherapy or radiotherapy might facilitate effector T cell infiltration by eliciting tumor tissue to release more antigens (52, 54). Another actionable approach involves the use of neoantigen-specific immunotherapy and PD-1 or PD-L1 inhibitor therapy. The rationale for this combination is that blocking active checkpoints might reactivate T cell function (70). Furthermore, the strategy combining molecularly targeted therapy with immunotherapy has been studied in patients with melanoma, and a synergistic effect has been observed (71). These combination strategies may be the potential future directions in the treatment of EC.

As an emerging therapeutic approach, neoantigen-specific immunotherapy has shown potential therapeutic effect on EC in several preclinical and clinical trials. Here, we provide a clear picture of the clinical application of neoantigens in EC from tumor-specific cancer vaccines and adoptive T cells to combination therapy. We also discuss the current barriers and strategies, thus providing direction for future investigation.

## Author Contributions

L-QC and YY conceptualized the study, revised the manuscript, and supervised the study. Y-MG and YZ conceptualized the study, drafted the manuscript, and made the figures. Y-MG and YZ collected the literature and revised the manuscript. All authors contributed to the article and approved the submitted version.

## Funding

This study was supported by the National Natural Science Foundation of China (Grant No. 81970481) and 1.3.5 project for disciplines of excellence-Clinical Research Incubation Project, West China Hospital, Sichuan University (Grant Nos. 2020HXFH047 and 20HXJS005).

## Conflict of Interest

The authors declare that the research was conducted in the absence of any commercial or financial relationships that could be construed as a potential conflict of interest.

## Publisher’s Note

All claims expressed in this article are solely those of the authors and do not necessarily represent those of their affiliated organizations, or those of the publisher, the editors and the reviewers. Any product that may be evaluated in this article, or claim that may be made by its manufacturer, is not guaranteed or endorsed by the publisher.
